# Acquired constriction ring syndrome as a cause of inconsolable cry in a child: a case report

**DOI:** 10.1186/1757-1626-1-92

**Published:** 2008-08-14

**Authors:** Vinay Singh, Pankaj Singh, Amit Sharma, Jay Sarkar

**Affiliations:** 1 Registrar Trauma & Orthopaedics Surgery, Luton and Dunstable Hospital NHS, Foundation Trust, Lewsey Road, Luton, LU4 0DZ, UK; 2 Registrar Gamma Knife Radiosurgery, Royal Hallamshire Hospital, Sheffield, S10 2JF, UK; 3 Consultant Foot and Ankle Surgeon, Luton and Dunstable Hospital NHS, Foundation Trust, Lewsey Road, Luton, LU4 0DZ, UK

## Abstract

Acute constriction ring syndrome (ACRS) is a rare clinical condition characterized by formation of a circumferential constriction ring around an appendage or genitalia. Cases are mostly reported in infants and young children. Early recognition and a definitive treatment are of paramount importance in order to avoid irreversible ischemia and possible auto-amputation. We describe a case of a 14-month-old child presented to casualty with a history of refusal to feed and inconsolable cry. Parents noticed a recent swelling of left third toe. On careful examination the child was found to have an acquired constriction ring secondary to a tightly wrapped hair around left third toe. An urgent surgical decompression was done by the orthopaedic team with complete resolution of symptoms. We summarized the pathophysiology of ACRS underlining the need of awareness in treating physicians. The possible medico legal implications should be kept in mind bearing a suggested link with non-accidental injury.

## Background

Acquired constriction ring syndrome (ACRS) is more commonly known as hair tourniquet syndrome or hair thread tourniquet syndrome [1]. It is clinical condition characterized by circumferential constriction of an appendage or genitalia by human hair, synthetic fibre or a thread [2]. It usually affects infants and has infrequently involved adolescent and cognitively impaired adults [3,4]. Congenital, [1,5] accidental as well as non-accidental [6-8] aetiologies have been proposed by different authors. Lesser toes remain the most common site affected^1 ^followed by fingers and genitalia [8-11].

## Case report

**Figure 1:**
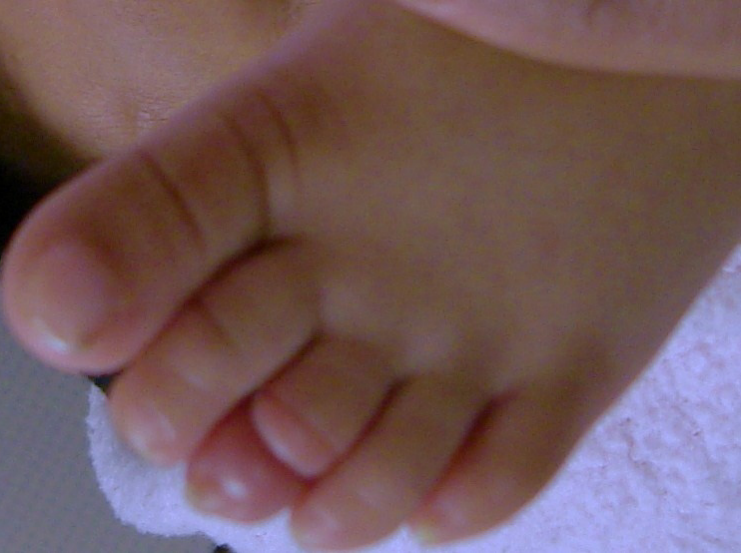
The constriction ring of third toe can be seen clearly at the level of distal inter-phalangeal joint with redness and oedema.

**Figure 2:**
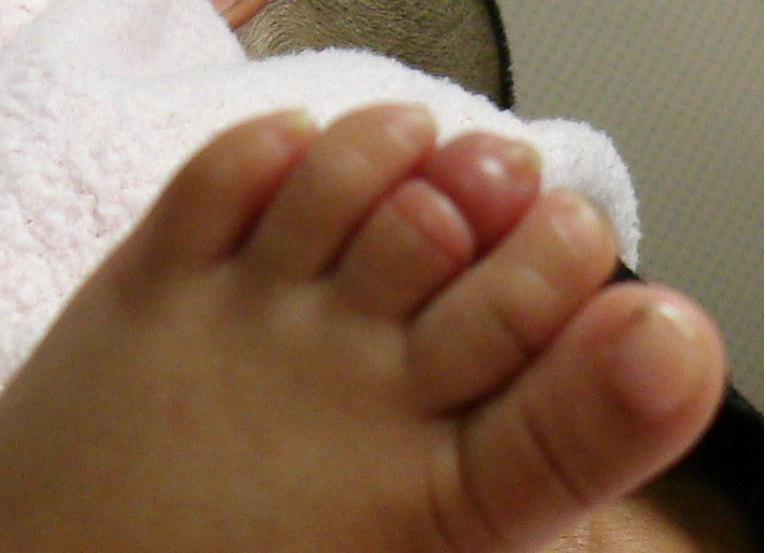
The constriction ring is visible in another image with resultant distal oedema and swelling.

A 14-months-old baby girl was brought to casualty by mother with an inconsolable cry and refusal to feed in our institute just after Christmas in 2007. Mother noticed a swelling and redness of left third toe while changing her socks in the morning. There was no history of trauma or any congenital deformity. On examination child was afebrile and systemically well. The digit was tender on touch with profound redness and swelling involving the distal phalange. The initial naked-eye examination done in emergency department failed to reveal a definitive cause. The child was referred to orthopaedic team for further opinion. The extensive swelling in an already distressed child was restricting the extent of assessment. Appropriate analgesics were given after establishing an intravenous access. A lens and loupe examination performed in emergency treatment room under light sedation, revealed the presence of a constriction ring at distal inter-phalangeal joint (Fig 1, 2). The skin was breached on dorsal surface of toe with volar surface showing a deeply buried ring of hair. Delayed capillary refill indicated an imminent danger of gangrene secondary to ACRS. The examination of other digits and genitalia was unremarkable. A non-accidental cause was ruled out with the help of paediatricians.

**Figure 3:**
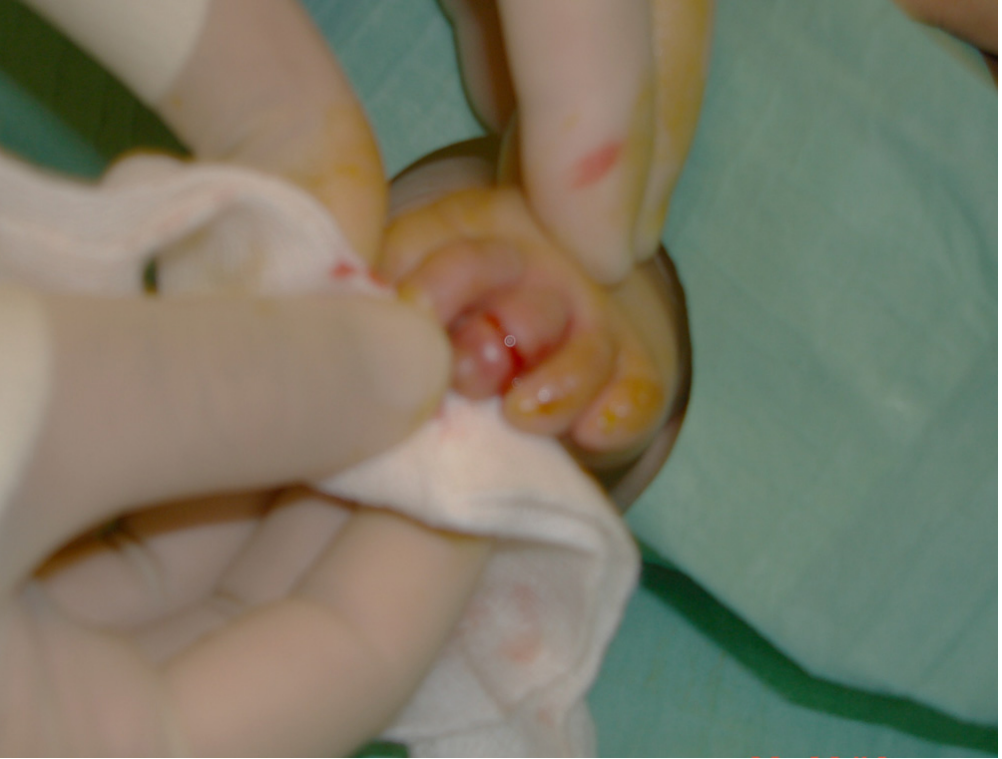
Intraoperative photograph taken after the release of constriction ring. Note the releasing incision at the dorsal surface.

Child was urgently taken to theatre to avoid further neurovascular compromise. Constriction ring of hair was removed with a pair of fine scissors under general anaesthetic (Fig 3). Hair was examined for the presence of well formed knots. Constriction ring was also decompressed by a small dorsal slit-incision down to the bone avoiding extensor tendons and the wound was left open. The child was comfortable in the postoperative period with a significant reduction of swelling by the next day. Prophylactic oral antibiotics were given for a week to avoid secondary infection. The kid was discharged from clinic in a week's time after complete resolution of symptoms.

## Discussion

Hair tourniquet syndrome is characterized by circumferential strangulation of an appendages or genitalia by human hairs or fibres. The condition was first described in 1832 [12] and the term hair tourniquet syndrome was first coined by Quinn in 1971[13]. Since then various authors have used eponyms hair thread tourniquet syndrome and acquired constriction ring syndrome to describe the same entity. The exact incidence of the condition is not known but mostly considered to be rare [2]. Whereas there are reports in paediatric, emergency and orthopaedic subspecialty periodicals, the condition failed to attract the attention of general orthropods [2]. The condition primarily involves infants with over 80% of the cases being reported in a population younger than 2 months [1]. However, there are occasional reports of adolescents and adults with cognitive impairment being involved [3,4]. Hair remains the most common causative agent with a reported incidence of 79% in one study [14]. Hair has unique physical characteristics which make it an ideal tourniquet. It is thin, elastic and expansible when wet while constricts as it dries off without losing its tensile strength [1].

Etiology of ACRS remains a matter of debate with more then one mechanisms playing role including accidental, [1,2] non-accidental [6-8] and congenital [1,5]. There are some predisposing conditions described in literature indicating the accidental nature. Baby cloths such as mittens and single piece jumpsuits may cause accumulation of hairs and may pose increase risk of ACRS [1,2]. Postpartum excessive hair loss (telogen-effluvium) has been known to predispose risk of ACRS [14,15]. In spite of the name acquired constriction, a group of authors believe ACRS to be of congenital origin [1,5] and the high incidence (> 80%) of cases in early infantile life has been attributed to its congenital origin. The other group, however strongly advocate ARCS to be non-accidental in nature until proven otherwise [6-8]. Authors suggested that non-accidental injury (NAI) to be considered where there is no reasonable explanation for presence of meticulously wrapped constriction ring or presence of well formed knots [5]. ACRS involving limb are mostly accidental and that involving genitals are thought to be associated with NAI [15]. The absence of well formed knots and an unremarkable paediatric assessment effectively ruled out NAI in our case. Contrary to digital variety the genital cases are usually seen in relatively older children ranging from 4–11 years. This age corresponds to the Sigmund Freud's phallic (or clitoral) stage of psychosexual development when kids start toying with their genitalia out of curiosity. The application of a tourniquet around genitalia may be a possible reflection of this psychosexual tendency. Some of the cultural practices also warrant a mention where an intentional tourniquet applied to ward off evil spirits or treat urinary incontinence and nocturnal emissions led to ACRS [8]. The pathophysiology of ACRS can be compared with compartment syndrome. The constriction ring interferes with the distal venous and lymphatic drainage at first, leading to venous engorgement. This progressive oedema further exacerbates the constriction setting a vicious circle ultimately ushering the ischemia and gangrene if not relieved in time [6].

Different measures have been suggested to prevent the ACRS. Counselling of postpartum mother is imperative and crucial. It is important to educate her to perform through regular checks of limbs to ensure that no hairs are entangled around fingers and toes of the baby. Mittens, single piece jumpsuits and clothing covering fingers and toes should be regularly checked for presence of loose hairs and should be washed inside out [14].

Early recognition with an urgent decompression remains the mainstay of treatment in an established case. Apart from the affected toe, a thorough examination should include all unaffected toes, fingers and genitals in order to rule out simultaneous involvement else where. Decompression may be carried out with fine scissors or using a depilatory cream in initial stages of ACRS when skin is intact and swelling is minimal [1,2]. In advanced stages when swelling is profuse or skin has breached, then it is advisable to carry out urgent complete decompression in theatre preferably under general anaesthetic [2]. Adequate light and magnification will further aid the search for constricting agent which may be lying buried deeply inside the subcutaneous tissues. The skin may re-epithelialize over the buried hair making the exploration further difficult [2]. Once the constricting agent is completely removed the soft tissue constriction ring itself should be decompressed by making a small vertical bone deep incision on dorsal surface avoiding extensor tendons [2]. It is vital to examine the tourniquet for presence of a well formed knot as it would strongly suggest presence of NAI. Decompression in casualty may be performed in early stages of ACRS but must be avoided if skin has breached or there is profound swelling making adequate visualization of constricting agent impossible. Inadequate visualization in fully established ACRS may lead to incomplete release and adverse clinical consequences.

## Conclusion

Parental education is of immense value in reducing the incidence of ACRS. For clinicians, it is crucial to treat ACRS as an appendage threatening emergency and perform urgent complete decompression in theatre. Incomplete decompression may lead to further ischemia and auto-amputation. Inconsolable cry and refusal to feed are two most common but equally no-specific symptoms in infants and young children. Clinicians working in acute paediatric settings should be aware of the entity of acquired constriction ring syndrome as a possible cause, after excluding the common ones. NAI should be kept in mind when dealing with ACRS and through examination for presence of knots at time of decompression is important as it may give precious clue to etiology. ARCS, a potentially reversible cause of neurovascular compromise, should be kept in differential diagnosis of acute swelling in an appendage or genitalia of an obscure etiology.

## Abbreviations

ACRS: Acquired constriction ring syndrome

## Competing interests

The authors declare that they have no competing interests.

## Authors' contributions

VS: Operating surgeon, collected clinical details including photographs, summarised the case history and prepared first draft. PS:Conducted a literature search, design and formatting of final manuscript, including grammar. Verified the authenticity of scientific content. AS: Helped in conducting the literature search and extracting the papers from library and internet. He also contribute in preparation of electronic images and electronic formatting of manuscript. JS: Treating consultant and performed final editing of the manuscript.

## Consent

A fully informed written consent was obtained from the patient for the publication of this case report and accompanying images. A copy of the written consent is available for review by the Editor-in-Chief of this journal.

## References

[B1] O'Quinn JC, Friedman RL, Wilms KL (2006). Acquired constriction ring syndrome. J Am Podiatr Med Assoc.

[B2] Haene RA, Loeffler M (2007). Hair tourniquet syndrome in an infant. J Bone Joint Surg Br.

[B3] Bacon JL, Burgis JT (2005). Hair tourniquet syndrome in adolescents: a presentation and review of literature. J Paediatr Adolesc Gynecol.

[B4] Miller RR, Baker WE, Brandeis GH (2004). Hair-thread tourniquet syndrome in a cognitively impaired nursing home resident. Adv Skin Wound Care.

[B5] Sudhan ST, Gupta S, Pluttarco C (2000). Toe-tourniquet syndrome-accidental or intentional?. Eur J Pediatr.

[B6] Klusmann A, Lenard HG (2004). Tourniquet syndrome – accident or abuse?. Eur J Pediatr.

[B7] Sylwestrzak MS, Fischer BF, Fischer H (2000). Recurrent clitoral tourniquet syndrome. Pediatrics.

[B8] Rich MA, Keating MA (1999). Hair tourniquet syndrome of the clitoris. J Urol.

[B9] Alverson B (2007). A genital hair tourniquet in a 9-year-old girl. Pediatr Emerg Care.

[B10] Garty BZ, Mimouni M, Varsano I (1983). Penile tourniquet syndrome. Cutis.

[B11] Haddad FS (1982). Penile strangulation by human hair. Report of three cases and review of literature. Uorl Int.

[B12] Dr G (1832). Ligature of the penis. Lancet.

[B13] Quinn NJ (1971). Toe tourniquet syndrome. Pediatrics.

[B14] Strahlman RS (2003). Toe tourniquet syndrome in association with maternal hair loss. Pediatrics.

[B15] Lohana P, Vashishta GN, Price N (2006). Toe-tourniquet syndrome: a diagnostic dilemma!. Ann R Coll Surg Engl.

